# Suicidal Ideation in Newly-Diagnosed Chinese Cancer Patients

**DOI:** 10.3389/fpsyt.2020.00708

**Published:** 2020-07-23

**Authors:** Yongfu Zhang, Wengao Li, Zijun Zhang, Hengwen Sun, Samradhvi Garg, Yuan Yang, Hongmei Wang

**Affiliations:** ^1^ Department of Anesthesiology, Guangzhou Women and Children’s Medical Centre, Guangzhou, China; ^2^ Department of Psychiatry, Southern Medical University Nanfang Hospital, Guangzhou, China; ^3^ Social Work and Counselling Team, CNSST Foundation, Auckland, New Zealand; ^4^ Department of Radiotherapy, Cancer Center, Guangdong Provincial People’s Hospital (Guangdong Academy of Medical Sciences), Guangzhou, China; ^5^ School of Health in Social Science, University of Edinburgh, Edinburgh, United Kingdom; ^6^ Department of Radiation Oncology, Southern Medical University Nanfang Hospital, Guangzhou, China

**Keywords:** cancer, China, factors, prevalence, suicidal ideation

## Abstract

**Objective:**

Suicide is one of the main reasons cited behind the death rate of cancer, and suicidal ideation (SI) is the first step toward it. This study aimed to investigate the prevalence and associates of suicidal ideation in newly-diagnosed Chinese cancer patients.

**Methods:**

This multicenter study was conducted from January 2018 to September 2019. Eligible participants were asked to complete a Case Record Form (CRF), the Patient Health Questionnaire (PHQ-9), General Anxiety Disorder Questionnaire (GAD-7), Fear of Cancer Recurrence Questionnaire (FCRQ-7), and McGill Pain Questionnaire-Visual Analogue Scale (MPQ-VAS). Univariate analyses and multivariate logistic regression analyses were conducted for assessment.

**Results:**

Out of 603 patients, 91 (15.1%, 95%CI: 12.23%–17.96%) reported suicidal ideation in the last 2 weeks. Physical comorbidities (OR=1.808, P=0.039), childhood adversity experience (OR=5.999, P=0.001), cancer pain (OR=1.828, P=0.047), depression (OR=2.811, P=0.013), and anxiety (OR=6.532, P<0.001) were significantly associated with suicidal ideation. It was also found that patients who regularly exercised were less likely to report suicidal thoughts (OR=0.172, P=0.007).

**Conclusion:**

Physical comorbidities, body ache, and mood disturbances are possible risk factors for suicidal ideation that warrant further attention in clinical practice. Preventive measures, such as systematic screening and arrangement for regular check-ups, could be beneficial to lower the risk of suicide.

## Introduction

Emotional distress is considered as the sixth vital sign targeting a cancer patient’s well-being along with signs of respiration, body temperature, blood pressure, heart rate, and pain (1). With the growing awareness and pivotal attention being paid to mental health, a number of researchers in the field of oncology have focused on cancer patients’ psychological wellbeing. These have then, subsequently reported a high prevalence of depression, anxiety, and suicidality as a part of it (2–6). Suicide is one of the main reasons subscribed behind the death rate affecting cancer. Spoletini et al. found a higher risk of suicide in cancer patients compared to the general population (7), and Anguiano et al. reported that there is a “suicide peak” in the first year after initial cancer diagnosis (8). Newly-diagnosed cancer patients have unique biological and psychological needs. Therefore, more efforts should be made to provide better insights into this population’s well-being. It is of significance to identify the pattern for suicidal ideation and its correlates in this population to further help clinicians adopt a more appropriate strategy and interventions to reduce the risk of suicide.

Suicidal ideation is commonly recognized as the first step, thought, and plan toward suicide (9, 10). A recent review reported a wider range of percentages between 0.8 and 71.4% of suicidal ideation in cancer patients, compared to a range of 1.1–19.8% in the general population (11). Another review, involving 44 relevant studies indicated that the prevalence of suicidal ideation ranged from 0.8 to 46.2% among cancer patients; with lowest figures being reported by an American study while the highest percentage was reported in Chinese samples (12).

There have been a variety of sociodemographic, clinical, and psychological variables found to be related to suicidal ideation. For example, advanced cancer stage, impaired physical functioning, cancer pain, depression, anxiety, hopelessness, existential distress, and limited social support were consistently proven to be risk factors for suicidal ideation in cancer patients (11). Gender, age, race, and treatment type were also reported to be associated with suicidal ideation, however, the findings have been often conflictive (12). A recent Chinese study reported that depression, anxiety, metastatic cancer, poor performance status, surgery, and palliative care were significantly associated with suicidal ideation, whereas, compared to demographic or clinical factors, psychological characteristics were more vital in predicting patient’s suicidal ideation (13).

It has been shown that suicidal ideation could lead to suicidal behavior and a completed suicide as one of the factors contributing to the high mortality rate behind cancer (14). However, most existing studies on suicidal ideation have been performed in cancer samples with mixed-time since diagnosis. To the best of our knowledge, evidence-based assessments targeting newly-diagnosed cancer patients are scarce. Moreover, the first few months after a confirmed cancer diagnosis is a critical time-window for patients because of the changes in both their physiology and psychological well-being. Therefore, before developing preventive strategies and alleviating the negative outcomes of suicidal ideation for this vulnerable population, it is important to understand its epidemiology and its correlates. Thus, the current study aims to provide an evaluation of the prevalence and correlates of suicidal ideation among newly-diagnosed (time lapse since diagnosis ≤ 6 months) Chinese cancer patients.

## Methods

### Participants and Study Procedure

This is a multicenter, cross-sectional study conducted in southern China. A consecutive sampling method was utilized to recruit patients from Guangdong Provincial People’s Hospital (Cancer Centre), Guangzhou Women and Children’s Medical Centre (Breast Cancer Centre), and Southern Medical University Nanfang Hospital (Department of Radiotherapy). Data was collected from January 2018 till September 2019. To be included in the study, participants needed to: 1) be above 18 years old; 2) have had a cancer diagnosis within 6 months; 3) be able to read and write Mandarin and/or Cantonese; and 4) provide written informed consent. Simultaneously, participants were excluded if they had disturbance of consciousness, or were blind or deaf.

All patients were approached in the waiting areas of these above mentioned facilities by trained research nurses who attempted to explain the study’s aim and procedure. For those then found eligible, and could provide written informed consent form; a face-to-face interview was conducted by a senior physician who was responsible for on-site recruitment and assessment. Participants were assured that the total interview time would not exceed 10 min and that they would not have the appointments delayed with their oncologist either. Participants were asked to complete a personal information sheet and a set of standardized questionnaires for assessment. All participants were assured that their participation would be kept confidential, and they could withdraw participation at any given time. The recruitment process was supervised by a consultant oncologist (HMW). Ethical approvals were obtained from participating hospitals (ref No: NFEC-2018-038).

### Measurements

#### Demographic and Clinical Data

A Case Record Form (CRF) was designed for this study to collect generic demographic and clinical information, such as gender, age, education level, marital status, employment status, personal monthly income, treatment/family history, physical comorbidities, lifestyle (smoking and exercise), and previous experience. Experience of adversity in childhood and experience of severe illness were assessed by two yes/no questions (15): 1) Have you ever experienced any childhood adversity, such as sexual abuse, bullying, traffic accident, or natural calamities? 2) Have you ever experienced any severe illness during childhood, such as childhood cancer, or traumatic injury? Consequently, stress was assessed by a single-item question (16): what is your current stress level? For which four responses were provided, namely: none, mild, moderate, and high level.

#### Depressive Symptoms

Patient’s depressive symptoms were assessed by the Patient Health Questionnaire (PHQ). It is a nine-item self-report measure and is commonly used in medical settings. Its response anchors a range from 0 to 3, and a total score of 5 or more indicates a prevalence of depressive symptoms (17). The Chinese version of PHQ-9 shows good psychometric properties, of which the Cronbach’s alpha is 0.89 (18).

#### Anxiety Symptoms

The General Anxiety Disorder Questionnaire (GAD) is a seven-item self-report scale used to measure an individual’s anxiety symptoms. Response options available are rated from 0 to 3; and a total score of 5 or more indicates a dominance of anxiety symptoms (19). The GAD-7 has been translated and well-validated in Chinese language. The Chinese version of GAD-7 has an internal consistency of 0.91 (20).

#### Fear of Cancer Recurrence

The seven-item Fear of Cancer Recurrence Questionnaire (FCRQ-7) is used to assess patient’s recurrence fears and has been used with patients riddled with breast, colorectal, head, and neck cancer in a variety of clinical centers in the UK (21). The reliability of this questionnaire is good with an internal consistency of 0.92 (95%CI: 0.90, 0.94) with evidence for validity (22). The statistical 60th (score=17) and 90th (score=27) percentiles are regarded as levels for “moderate” and “high” reports of patient’s FCR respectively. The Chinese version of FCRQ-7 shows good psychometric properties, of which the Cronbach’s alpha is 0.87 (23).

#### Cancer Pain and Suicidal Ideation

The McGill Pain Questionnaire-Visual Analogue Scale (MPQ-VAS) is one of the most widely used tests for the measurement of pain, and it has been validated into the Chinese language in 2013 (24). The score of VAS ranges from 0 (no pain) to 100 (worst possible pain) (25). In the current study, participants were first asked: 1) Are you currently in any pain from cancer? (yes/no question), then a VAS was provided to further identify the exact pain severity thus reported. Patient’s suicidal ideation was assessed by a standardized yes/no question adapted from Kessler’s question (26, 27): Have you ever thought about killing yourself in the last 2 weeks? For those who answered “yes” to this question, they were classified under “Current Suicidality”.

### Statistical Analyses

All data analyses were performed by SPSS Version 24.0. Descriptive statistics were utilized to characterize all study variables. Normal distribution assumption was checked by one-sample Kolmogorov-Smirnov test. Sociodemographic and clinical variables between the two groups were investigated using independent sample t-test (for normally distributed continuous data), chi-square test (for categorical variables), or Mann-Whitney U test (for non-normally distributed continuous data). All variables that were found to be significant in the univariate analyses were further tested by multivariate logistic regression with “enter” method. Suicidal ideation was allocated as the dependent variable, while those with significant group differences in the above univariate analyses were entered as independent variables. Significance was set at 0.05, with two-tailed tests.

## Results

### Patient Characteristics

A total of 603 patients agreed to participate, and completed the survey. The mean age of patients was 47.72 (SD=11.49) years. Simultaneously, around 90% (n=543) of the participants were female, and 78.5% (N=473) of the participants were specifically diagnosed with breast cancer. Most of the patients were married (85.2%), with low education level (high school or below=67.7%), had previously received surgery (92.9%), chemotherapy (86.1%), and radiation treatment (87.4%). Of the 603 patients, 91 (15.1%, 95%CI: 12.23%–17.96%) reported suicidal ideation in the last 2 weeks.

### Univariate Analyses

In univariate analyses, suicidal ideation was significantly associated with patient’s age (P=0.022), marital status (P=0.007), radiotherapy (P=0.025), cancer pain (P=0.038), physical co-morbidity (P=0.038), exercise (P<0.001), childhood adversity experience (P=0.001), and current stress levels (P<0.001). Those who were younger, single, had received prior radiation treatment, in current cancer pain, with antecedent childhood adversity experience, other physical co-morbidities and under high stress levels were more likely to have suicidal ideation than their counterparts. Depression, anxiety, and fear of cancer recurrence symptoms were also significantly associated with suicidal ideation (P all <0.001). Controversially, patients who kept routine exercise plans (more than 60 min/d) were less likely to report suicidal ideation than those without physical exercise (P<0.001). Patient’s demographic, clinical, as well as psychological characteristics are presented in **Table 1**.

**Table 1:** Factors associated with suicidal ideation (N=603).

Variable	Suicidal Ideation					
	Total (%)	No (%)	Yes (%)	X^2^/Z	df	P
**Gender**						
Male	60 (10.0)	51 (85.0)	9 (15.0)	0.001	1	0.983
Female	543 (90.0)	461 (84.9)	82 (15.1)			
**Cancer site**						
Breast	473 (78.4)	408 (86.3)	65 (13.7)	3.286	3	0.350
Lung	65 (10.8)	53 (81.5)	12 (18.5)			
Colorectal	49 (8.1)	39 (79.6)	10 (20.4)			
Nasopharynx	16 (2.7)	12 (75.0)	4 (25.0)			
**Cancer stage**						
Stage 1	43 (7.1)	38 (88.4)	5 (11.6)	2.871	3	0.412
Stage 2	264 (43.8)	225 (85.2)	39 (14.8)			
Stage 3	243 (40.3)	208 (85.6)	35 (14.4)			
Stage 4	53 (8.8)	41 (77.4)	12 (22.6)			
**Marital status**						
Single	47 (7.8)	32 (68.1)	15 (31.9)	12.137	3	**0.007**
Married	514 (85.2)	442 (86.0)	72 (14.0)			
Divorced	25 (4.1)	24 (96.0)	1 (4.0)			
Widowed	17 (2.8)	14 (82.4)	3 (17.6)			
**Education level**						
High School or below	408 (67.7)	350 (85.8)	58 (14.2)	1.297	2	0.523
Undergraduate	173 (28.7)	145 (83.8)	28 (16.2)			
Postgraduate or above	22 (3.6)	17 (77.3)	5 (22.7)			
**Living condition**						
Living Alone	27 (4.5)	23 (85.2)	4 (14.8)	0.093	2	0.955
Living with Families	560 (92.9)	475 (84.8)	85 (15.2)			
Living with Friends	16 (2.7)	14 (87.5)	2 (12.5)			
**Employment**						
Full Time	236 (39.1)	204 (86.4)	32 (13.6)	2.563	3	0.464
Part Time	36 (6.0)	30 (83.3)	6 (16.7)			
Unemployment	166 (27.5)	135 (81.3)	31 (18.7)			
Retired	165 (27.4)	143 (86.7)	22 (13.3)			
**Monthly salary (Yuan)**						
<3,000	260 (43.1)	216 (83.1)	44 (16.9)	3.181	3	0.365
3,000–5,000	166 (27.5)	143 (86.1)	23 (13.9)			
5,001–10,000	125 (20.7)	105 (84.0)	20 (16.0)			
>10,000	52 (8.6)	48 (92.3)	4 (7.7)			
**Surgery**						
No	43 (7.1)	36 (83.7)	7 (16.3)	0.051	1	0.821
Yes	560 (92.9)	476 (85.0)	84 (15.0)			
**Chemotherapy**						
No	84 (13.9)	70 (83.3)	14 (16.7)	0.189	1	0.664
Yes	519 (86.1)	442 (85.2)	77 (14.8)			
**Radiotherapy**						
No	76 (12.6)	58 (76.3)	18 (23.7)	5.011	1	**0.025**
Yes	527 (87.4)	454 (86.1)	73 (13.9)			
**Family cancer history**						
None	443 (73.5)	383 (86.5)	60 (13.5)	3.119	1	0.077
Yes	160 (26.5)	129 (80.6)	31 (19.4)			
**Physical comorbidity**						
None	383 (63.5)	334 (87.2)	49 (12.8)	4.324	1	**0.038**
Yes	220 (36.5)	178 (80.9)	42 (19.1)			
**Current smoker**						
No	575 (95.4)	489 (85.0)	86 (15.0)	–	-^a^	0.596
Yes	28 (4.6)	23 (82.1)	5 (17.9)			
**Daily exercise time**						
None	67 (11.1)	47 (70.1)	20 (29.9)	17.509	2	**<0.001**
<60 min	458 (76.0)	391 (85.4)	67 (14.6)			
>60 min	78 (12.9)	74 (94.9)	4 (5.1)			
**Childhood adversity exp**						
None	573 (95.0)	494 (86.2)	79 (13.8)	–	-a	**0.001**
Yes	30 (5.0)	18 (60.0)	12 (40.0)			
**Childhood severe illness exp**						
None	550 (91.2)	468 (85.1)	82 (14.9)	0.162	1	0.687
Yes	53 (8.8)	44 (83.0)	9 (17.0)			
**Current stress level**						
None	186 (30.8)	173 (93.0)	13 (7.0)	24.997	3	**<0.001**
Mild	238 (39.5)	199 (83.6)	39 (16.4)			
Moderate	134 (22.2)	111 (82.8)	23 (17.2)			
High	45 (7.5)	29 (64.4)	16 (35.6)			
	**M (SD)**	**M (SD)**	**M (SD)**	**T/Z**	**df**	***P***
**Age (years)**	47.72 (11.49)	48.18 (11.07)	45.19 (13.44)	2.294	601	**0.022**
**Cancer pain (VAS score)**	23.55 (17.03)	22.95 (16.67)	26.92 (18.66)	−2.072	- ^b^	**0.038**
**Depression (PHQ score)**	4.97 (4.80)	3.93 (3.61)	10.81 (6.27)	−10.306	- ^b^	**<0.001**
**Anxiety (GAD score)**	3.76 (4.24)	2.89 (3.47)	8.63 (4.82)	−10.711	- ^b^	**<0.001**
**Fear of recurrence (FCR score)**	19.85 (6.38)	19.07 (6.03)	24.22 (6.56)	−7.408	601	**<0.001**

in bold: P<0.05; a: Fisher’s exact test; b: Mann-Whitney U test.

Exp, experience; VAS, McGill Pain Questionnaire-Visual Analogue Scale; PHQ, Patient Health Questionnaire; GAD, Generalized Anxiety Disorder seven-item scale; FCR, fear of cancer recurrence; M, mean; SD, standardized deviation.

### Multivariate Logistic Regression Analyses

Multivariate logistic regression analyses confirmed that physical comorbidities (OR=1.808, 95%CI: 1.030–3.172, P=0.039), childhood adversity experience (OR=5.999, 95%CI: 2.095–17.177, P=0.001), cancer pain (OR=1.828, 95%CI: 1.009–3.310, P=0.047), depression (OR=2.811, 95%CI: 1.238–6.382, P=0.013), and anxiety (OR=6.532, 95%CI: 2.911–14.657, P<0.001) were significantly associated with a patient’s suicidal ideation. Additionally, patients who had routine exercise plans were less likely to report suicidal ideation (OR=0.172, 95%CI=0.048–0.619, P=0.007). Age, marital status, radiotherapy, stress level, and fear of cancer recurrence were no longer significant in multivariate logistic regression analyses (see **Table 2**).

**Table 2:** Multivariate logistic regression of factors associated with suicidal ideation.

Variable	Category	OR	95% CI Lower	95% CI Upper	df	P
**Age (years)**		0.978	0.954	1.003	1	0.079
**Marital status**	Single	ref	-	-	-	-
	Married	0.724	0.254	2.062	1	0.545
	Divorced	0.293	0.027	3.230	1	0.316
	Widowed	2.755	0.379	20.046	1	0.317
**Radiotherapy**		0.874	0.376	2.032	1	0.755
**Physical comorbidity**		1.808	1.030	3.172	1	**0.039**
**Daily exercise time**	None	ref	–	–	–	–
	Less Than 60min	0.360	0.171	0.757	1	**0.007**
	More Than 60min	0.172	0.048	0.619	1	**0.007**
**Childhood adversity exp**		5.999	2.095	17.177	1	**0.001**
**Current stress level**	None	ref	–	–	–	–
	Mild	1.701	0.764	3.786	1	0.193
	Moderate	1.384	0.580	3.300	1	0.464
	High	2.526	0.907	7.032	1	0.076
**Cancer pain**		1.828	1.009	3.310	1	**0.047**
**Depression**		2.811	1.238	6.382	1	**0.013**
**Anxiety**		6.532	2.911	14.657	1	**<0.001**
**Fear of recurrence**		1.080	0.477	2.443	1	0.854

CI: confidence Interval; in bold: P<0.05; Exp, Experience.

## Discussion

For newly-diagnosed cancer patients, the first few succeeding months after diagnosis could be overwhelming, scary, and lonely. However, there is a paucity of evidence regarding the unmet mental health needs of newly-diagnosed cancer patients. To the best of our knowledge, this is the first study that examines the prevalence and correlates of suicidal ideation among newly-diagnosed cancer patients in China. Suicidal ideation in this current study was reported at an occurrence of 15.1%, which was similar to some; but not all studies. Zhong’s study with 517 Chinese cancer inpatients showed that 15.3% of the patients reported suicidal ideation (13); while Tang’s investigation indicated that the corresponding prevalence was 18.1% (28). However, Cheng et al. reported that 46.2% of the cancer participants reported suicidal ideation (29). The difference of estimates in suicidal ideation found could be partially explained by the differences in the study sample, measurement tools/questions, defined timeframe, and assessment method.

Our study showed a significant association between physical comorbidities and suicidal ideation. A previous research in prostate cancer patients reported that men with comorbidities tend to report more depressive symptoms and suicidal ideation compared to those without comorbidities (30). Similar studies also indicate that patients with more physical comorbidities reported elevated psychological disturbances as well (31, 32). It is possible that individuals with more comorbidities suffer from more physical discomfort, pain, fatigue, and psychological distress, which results in more suicidal ideation.

There is a plethora of evidence supporting the significant causational relationship between childhood traumatic experience and suicide in difference populations (33–35). It is possible that stressful traumatic experience could largely waste a patient’s mental energy and resources, which makes them more vulnerable to mental disorders (36). From the perspective of Joiner’s interpersonal theory (37), specific childhood trauma, such as severe neglect and rejection, can plant the seed for social isolation in an individual’s mind. These individuals will then build up a belief that there is no one that they live for or belong to. This absence of a sense of belonging may result in higher risk of suicide in these individuals.

In accordance with previous findings in studies conducted, patients experiencing cancer pain are at higher risk of reporting suicidal ideation and/or behavior (38) than those otherwise. The reason for the same might be associated with the secondary psychological distress and depression raised by cancer pain (38). Researchers also indicated that most suicides by cancer patients who are suffering from pain at a terminal stage tend to be more rational than being driven by under-diagnosed depression (39). This study’s finding highlighted the importance of providing adequate pain treatment which may help in better clinical advancements and a reduced suicide rate.

A strong association between depression and suicidal ideation has been identified in previous studies (2, 4, 29). Specifically, researchers found that having depression significantly predicted a higher risk of unnatural mortality, i.e. accident, and suicide in breast cancer patients (40). In fact, suicidal ideation has always been classified as one of the core diagnostic criteria and critical representations of major depressive disorder (41). Being female, having depression and suicidality are all closely connected (40, 42, 43), while even symptoms of depression; such as hopelessness, insomnia, loss of appetite, loneliness, fatigue, and low self-esteem greatly increase the risk of suicidal ideation, planning, and attempt (9). According to the interpersonal psychological model in suicidal behavior (37), traumatic feelings, such as painfulness, inconvenience, hopelessness, and feeling of burdensomeness can strengthen a patient’s suicidal desire continually.

Regarding the association mapped between anxiety and suicidal ideation, inconsistent results were reported. For example, Xin et al. reported that compared to non-anxious group, patients with anxiety disorder were at a higher suicidality risk (44), while Xu et al. revealed that anxiety did not significantly predict cancer patient’s suicidal ideation (45). Therefore, owning to this cross-sectional design, the causality between anxiety, depression, and suicidal ideation could not be determined in the current study. There is a need for further longitudinal investigation to better examine the link between them.

Our results show a significantly negative association between exercising frequency and suicidal ideation. Cancer patients with regular exercise plans are less likely to have suicidal thoughts. A previous review, including 14 studies indicated that exercise was negatively associated with suicidal ideation in adults (46). Asthenia, weakness, and reduction in motility are crucial indicators in a positive diagnosis of depression (41). The negative link between exercise and suicide ideation could be mediated by depression. In addition, the protective influence of exercise on suicidality might also be related to stronger immunity and positive attitude toward life brought on by scheduled exercise.

There are several limitations that should be acknowledged in the current study. First, although, there was a large sample scale, the distribution of sample; in gender and cancer type was uneven. Most of the participants were females, and diagnosed with breast cancer. These cases could be substantial enough to cause a bias in the data, thereby, affecting the research findings and making them non-generalizable for the entire Chinese cancer population. Second, the results of our study were all based on self-rated inventory; recall bias might exist. A single item question was used to assess patient’s suicidal ideation. This item might be effective for assessment of explicit suicidal ideation but might fail to measure implicit ideation. Similarly, several factors, such as childhood adversity experiences and illness experiences were also assessed by single yes/no question, and these may not hold enough statistical power. Further studies with larger sample sizes, using validated objective instruments are warranted to further attest to these claims. Thirdly, other variables associated with suicidal ideation, such as time lapse since diagnoses, and pre-existing mental disorders, were not examined in this study. The mediation effect of emotional disturbances (such as, depression and anxiety) in determining suicidal ideation was not explored in detail either. Lastly, this was a cross-sectional study which could not draw any causal association between tested variables and suicidal ideation, hence, raising the need for further studies using longitudinal study design.

## Conclusion

In conclusion, physical comorbidities, body ache, and mood disturbances are possible risk factors for suicidal ideation that warrant further attention in clinical practice. Considering the significant risk for suicide for cancer riddled population; preventive measures, such as regular screening and effective pain management, psychological supports should be performed widely. Health policy makers and health professionals should improve early identification of high risk patients and provide easy access to counseling and psychotherapy services for those in need.

## Data Availability Statement

The raw data supporting the conclusions of this article will be made available by the authors, without undue reservation.

## Ethics Statement

The studies involving human participants were reviewed and approved by Nanfang Hospital Research Ethics Committee, Southern Medical University (ref No: NFEC-2018-038). The patients/participants provided their written informed consent to participate in this study.

## Author Contributions

Study design: YZ, YY, and HW. Data collection, analysis and interpretation: YZ, HS, and WL. Drafting of the manuscript: YZ, WL, and ZZ. Critical revision of the manuscript: SG, YY, and HW. All authors contributed to the article and approved the submitted version.

## Funding

This study is funded by the President Foundation of Nanfang Hospital, Southern Medical University (2017L001), and the Guangzhou Science and Technology Project (201804010132).

## Conflict of Interest

The authors declare that the research was conducted in the absence of any commercial or financial relationships that could be construed as a potential conflict of interest.
